# Cyclosporiasis Outbreak, Indonesia

**DOI:** 10.3201/eid1109.040947

**Published:** 2005-09

**Authors:** Marjolijn C.A. Blans, Ben U. Ridwan, Jaco J. Verweij, Maja Rozenberg-Arska, Jan Verhoef

**Affiliations:** * Gelre Ziekenhuizen, Apeldoorn, the Netherlands;; † University Medical Center Utrecht, Utrecht, the Netherlands;; ‡ Leiden University Medical Center, Leiden, the Netherlands

**Keywords:** Cyclospora cayetanensis, Indonesia, outbreak, dispatch

## Abstract

We describe an outbreak of *Cyclospora cayetanensis* infection among Dutch participants at a scientific meeting in September 2001 in Bogor, Indonesia. Fifty percent of the investigated participants were positive for *C. cayetanensis*. To our knowledge, this outbreak is the first caused by *C. cayetanensis* among susceptible persons in a disease-endemic area.

*Cyclospora cayetanensis* is a newly recognized protozoan parasite that causes gastrointestinal illness. *C. cayetanensis* infection is mostly characterized by a gradual onset of watery diarrhea, sometimes with explosive diarrhea, nausea, and abdominal cramping. Symptoms are often prolonged and can relapse after months (*1*). Oocysts of *C. cayetanensis* are, in comparison with those of other coccidian parasites, noninfectious in freshly excreted stool. Therefore, direct person-to-person transmission through fecal exposure is unlikely. Food and water contaminated with sporulated oocysts are the primary modes of transmission (*1*). Infections have been associated mainly with outbreaks from eating food such as raspberries, salads, and basil (*1**–**3*). Infections with *C. cayetanensis* are seasonal; in the tropics, the wet and cooler seasons provide conditions more favorable for sporulation than do the dry and warmer seasons (*1**,**3*).

A scientific meeting involving Dutch and Indonesian microbiologists was held September 2–6, 2001, in Indonesia. Immediately after the meeting, several participants reported mild-to-severe gastrointestinal symptoms, and *C. cayetanensis* infection was diagnosed in 4 participants. We investigated the extent of the outbreak among participants from our institute and identified *C. cayetanensis*–specific symptoms.

## The Study

The meeting was held in a hotel near Bogor, Indonesia. All Dutch participants stayed in this hotel during the meeting and consumed the same meals (buffet). After the meeting, approximately half of the participants went home, while others took the opportunity to travel further.

Six weeks after the meeting, all members of our institute who visited the Bogor meeting were asked to participate in a cohort study. Participants were asked to complete a questionnaire and deliver 2 fecal samples. The questionnaire was set up to collect information about gastrointestinal symptoms, duration of stay, and results of earlier fecal diagnostic examination.

One of the 2 fecal samples was directly examined for *C. cayetanensis* after Ridley concentration by 2 microscopy methods. Presence of *C. cayetanensis* was demonstrated by nonrefractile spheres seen in a direct saline wet mount or by light pink–to–deep red, 8- to 10-µm long oocysts seen on modified acid-stained smears. Because the aim of the study was to determine the extent and duration of *C. cayetanensis* infection in our cohort, no special efforts were made to detect other possible pathogens. Another fecal sample was stored at –20°C until DNA was isolated for polymerase chain reaction (PCR). After DNA isolation, *C. cayetanensis*–specific real-time PCR was performed as described previously (*4*). The specific primers and probe were based on the known small ribosomal subunit RNA gene sequence for *C. cayetanensis*. This real-time PCR was specific when tested with a range of other intestinal parasites, and the DNA of >0.5 oocysts was estimated as the detection limit. Names and clinical conditions of the participants were blinded to laboratory technicians.

A case-control study was set up to investigate whether certain gastrointestinal symptoms were associated with *C. cayetanensis* infection. A case was defined as *C. cayetanensis*–positive microscopic result, positive PCR result, positive *C. cayetanensis* diagnosis 1–6 weeks before entering the study, or any combination. Only data from participants who submitted completed questionnaires were used for statistical analyses.

Analyses were performed by using the χ^2^ test if variables were categorical. Continuous variables were not normally distributed; therefore, they were compared and tested with the Mann-Whitney *U* test. The ethics committee of the University Medical Center Utrecht approved the project; written informed consent was obtained from all participants before participation.

Thirty-two (94%) of the 34 attendees of our institute responded, and 29 completed the questionnaire; 3 participants delivered only fecal samples. Fourteen (48%) of the 29 attendees had cases that met the definition: 10 case-patients had a positive PCR result, 5 of which were also positive on microscopic analysis, and 4 case-patients had a positive diagnosis 1–6 weeks before entering the study. Fecal samples of these 4 case-patients were all negative on microscopic analysis; 2 were also negative on PCR analysis. Two samples were lost before PCR could be performed. Fecal samples from the 3 participants who did not return the questionnaire were all positive by PCR and negative by microscopic analysis. They were not included in the case-control study.

Symptoms of the 29 participants are listed in the Table. More women had cyclosporiasis than men (71% vs. 40%). Case-patients mentioned bowel disorders significantly more than noncase-patients: stomach cramps, nausea, and flatulence were common symptoms among the case-patients (71% vs. 40%, 93% vs. 27%, and 93% vs. 33%, respectively). Duration of symptoms was significantly longer for case-patients than for noncase-patients.

**Table:** Characteristics of meeting members exposed to *Cyclospora cayetanensis*, Bogor, Indonesia, 2001*

Variable	Cases (%), n = 14	Noncases (%), n = 15	p value†
Female sex	10 (71)	6 (40)	0.09
Symptoms			
Diarrhea‡	11 (79)	9 (60)	NS
Obstipation	3 (21)	0	NS
Stomach and abdominal cramps	10 (71)	6 (40)	0.09
Flatulence	13 (93)	5 (33)	0.001
Fever	2 (13)	1 (7)	NS
Nausea/appetite loss	13 (93)	4 (27)	<0.001
Median symptom duration (IQR), days§	41 (29–42)	1 (0–6)	<0.001
Median stay in Indonesia (IQR), days	14 (10.5–18.5)	16 (11–23)	NS

*NS, nonsignificant; IQR, interquartile range. †Determined by χ^2^ test for categorical variables and by Mann-Whitney *U* test for continuous variables. ‡Diarrhea was defined as >3 loose stools in 24 hours. §If patients had symptoms when entering the study, the duration was fixed at 42 days.

## Conclusions

We showed that approximately half of the investigated meeting attendees were positive for *C. cayetanensis*. The number of proven infected persons would be higher if the investigations had started directly after the first symptoms appeared. The fact that 6 weeks after the probable exposure, *C. cayetanensis* DNA was still detectable in 13 persons corresponds to the known persistence of the parasite. Diagnosis in our study was primarily based on PCR-positive results. PCR is a much more reliable method to detect *C. cayetanensis* than diagnostic microscopy (*4**,**5*); however, ultraviolet fluorescence microscopy may be as sensitive as PCR (*4*).

An outbreak with a common source of infection has not been proven. We could not investigate potential food sources because we started surveillance 6 weeks after the assumed exposure in Indonesia, when participants had returned to the Netherlands. Only 2 of the participants could recall a meal that might have been a source of infection, and each suggested a different meal. Participants may have acquired *C. cayetanensis* infection on other occasions during their stay in Indonesia. Genotyping the different isolates to connect cases on a molecular basis was not possible because the number of oocysts detected in stools was small. However, assuming a joint exposure to *C. cayetanensis* is not unreasonable. First and most important, 3 of the participants in this study went to the conference and returned immediately to the Netherlands, and the conference was the only place where infection may have been acquired. Secondly, infections were acquired during the season with low incidence of transmission, so a common source is a more obvious route of infection than cases acquired at separate occasions. In our study, the main symptoms of cyclosporiasis were stomach cramps, nausea, and flatulence, without bloody diarrhea. The prolonged and relapsing character of the symptoms, especially diarrhea and abdominal cramps, was striking. This pattern of symptoms is mentioned in other outbreaks as well and seems to be characteristic for cyclosporiasis (*1**–**3*). Although nonindigenous persons are at risk for travelers' diarrhea in Indonesia, we believe that the gastrointestinal problems in the patients in our study were caused by cyclosporiasis. All 4 patients with an earlier diagnosis of *C. cayetanensis* infection were successfully treated with cotrimoxazole.

In conclusion, we report a possible outbreak of *C. cayetanensis* among Dutch microbiologists attending a meeting in Indonesia. At least 50% of participants were infected. To our knowledge, this outbreak is the first of *C. cayetanensis* among susceptible persons in a disease-endemic area.
